# Supplementary material for: Placental DNA methylation changes and the early prediction of autism in full-term newborns
Source: PLoS One. 2021 Jul 14;16(7):e0253340. doi: 10.1371/journal.pone.0253340 (PMC8279352; doi:10.1371/journal.pone.0253340)
Supplement: S1 Table — (DOCX) [file pone.0253340.s001.docx]

**S1 Table:** Clinical and demographic characteristics: Full term Autism cases versus control subjects

|  | **Cases** | **Controls** | **p-value** |
| --- | --- | --- | --- |
| Number of subjects | 14 | 10 | n/a |
| Maternal age in years- Mean (SD) | 28.78 (5.3) | 27 (5.6) | 0.40 |
| Maternal race - n (%) | | | |
| White | 11 (78.5) | 9 (90) | 0.95 |
| African American | 1 (7.14) | 0 (0) |  |
| Other/Unknown | 2 (14.28) | 1 (10) |  |
| Gestational age (weeks) | 39.16 (1.04) | 39.25 (0.74) | 0.36 |
