# Supplementary material for: Placental DNA methylation changes and the early prediction of autism in full-term newborns
Source: PLoS One. 2021 Jul 14;16(7):e0253340. doi: 10.1371/journal.pone.0253340 (PMC8279352; doi:10.1371/journal.pone.0253340)
Supplement: S3 Table — A. Results of Term Autism Placenta based on intragenic CpGs only (with FDR p-value < 0.05). B. Results of Term Autism Placenta based on intergenic (nongenic) markers only (with FDR p-value < 0.05). (DOCX) [file pone.0253340.s003.docx]

**S3A Table:** Results of Term Autism Placenta based on intragenic CpGs only (with FDR p-value < 0.05).

|  | SVM | GLM | PAM | RF | LDA | DL |
| --- | --- | --- | --- | --- | --- | --- |
| AUC  95% CI | 0.9988  (0.9500-1) | 0.9978  (0.9500-1) | 0.9997  (0.9500-1) | 1.0000  (0.9900-1) | 0.9979  (0.9500-1) | 1.0000  (1-1) |
| Sensitivity | 0.9300 | 0.9200 | 1.0000 | 1.0000 | 0.9200 | 1.0000 |
| Specificity | 0.9400 | 0.9800 | 1.0000 | 1.0000 | 0.9600 | 1.0000 |

Important predictors in order:

SVM: cg01560871, cg18675381, cg23082393, cg07172793, cg27273946

GLM: cg23181591, cg19711553, cg08991599, cg11123972, cg24429450

PAM: cg18921306, cg24669932, cg06422189, cg18675381, cg11263351

RF: cg00225623, cg22557091, cg14410227, cg24151352, cg02469790

LDA: cg14225719, cg01268752, cg02478023, cg16930349, cg12769566

DL: cg23920016, cg18016370, cg24274662, cg16930349, cg21290550

**S3B Table:** Results of Term Autism Placenta based on intergenic (nongenic) markers only (with FDR p-value < 0.05).

|  | SVM | GLM | PAM | RF | LDA | DL |
| --- | --- | --- | --- | --- | --- | --- |
| AUC  95% CI | 0.9999  (0.9500-1) | 0.9989  (0.9500-1) | 0.9995  (0.9500-1) | 1.0000  (0.9900-1) | 0.9978  (0.9500-1) | 1.0000  (1-1) |
| Sensitivity | 0.9200 | 0.9500 | 1.0000 | 1.0000 | 0.9000 | 1.0000 |
| Specificity | 0.9300 | 0.9500 | 1.0000 | 1.0000 | 0.9500 | 1.0000 |

Important predictors in order:

SVM: cg21210224, cg12332415, cg20315257, cg05036212, cg23566374

GLM: cg27526774, cg19874314, cg01812571, cg01866955, cg10101463

PAM: cg23925650, cg23815825, cg09978533, cg25685359, cg07090401

RF: cg13443950, cg04285666, cg15853614, cg19631762, cg09452082

LDA: cg05036212, cg01812571, cg12332415, cg03139489, cg05090351

DL: cg05036212, cg01812571, cg24148133, cg02864638, cg03582285
