# Supplementary figures and images for: Placental DNA methylation changes and the early prediction of autism in full-term newborns
Source: PLoS One. 2021 Jul 14;16(7):e0253340. doi: 10.1371/journal.pone.0253340 (PMC8279352; doi:10.1371/journal.pone.0253340)

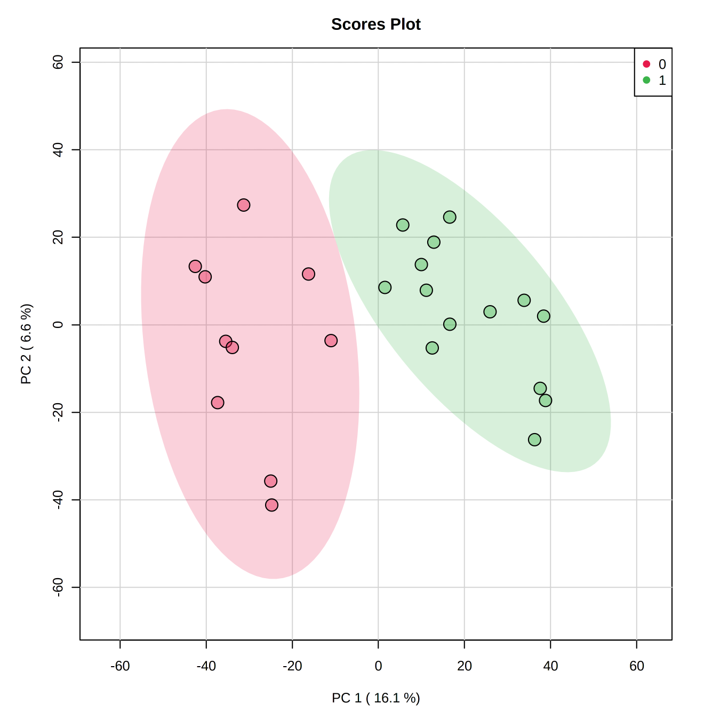

Supplement: S1 Fig — (TIF) [file pone.0253340.s004.tif]

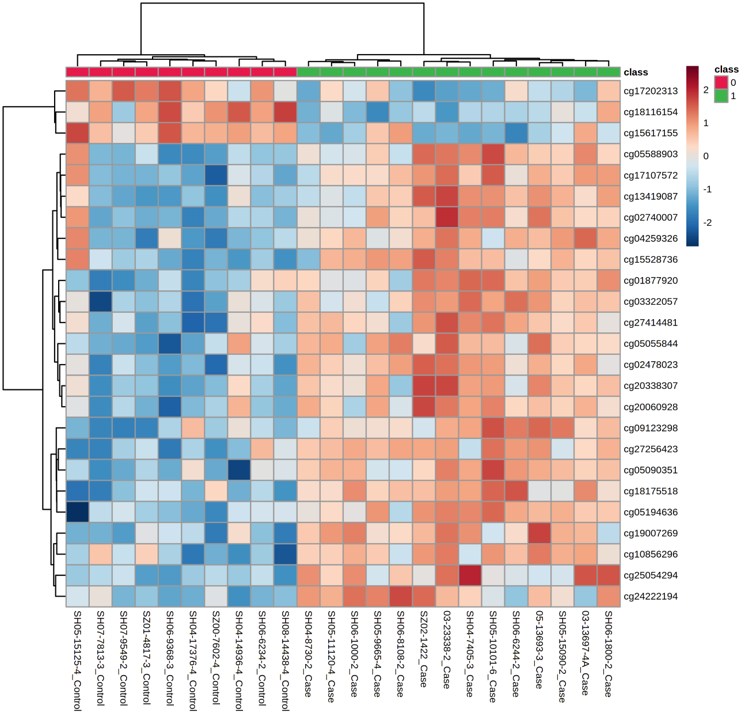

Supplement: S2 Fig — The hierarchical clustering showed separation of CpG markers based on hyper and hypomethylation status depicted on the figure completely methylated (red) to unmethylated (blue). (TIF) [file pone.0253340.s005.tif]
