# Supplementary material for: Placental DNA methylation changes and the early prediction of autism in full-term newborns
Source: PLoS One. 2021 Jul 14;16(7):e0253340. doi: 10.1371/journal.pone.0253340 (PMC8279352; doi:10.1371/journal.pone.0253340)
Supplement: Attachment — Submitted filename: 1-Response to Reviewers May 9 2021.docx [file pone.0253340.s006.docx]

Thank you for the opportunity to revise and resubmit our manuscript. We sincerely appreciate all your valuable comments and suggestions which have helped us improve the quality of the article. We believe that the revisions based on these comments have made this a better and clearer paper.

**Additional Editor Comments:**

Please provide answers to question raised by the reviewer. I want to add a few points for the improvement of this manuscript.

a) Please provide a pipeline figure for methods followed in this study.

**Author's Response:** Thank you for your comment. We have included the Schematics of the study design in the methodology section as fig 1.

b) Provide details of the pathway data in the result section and roles of these four pathways in autism.

**Author's response:** We have specified the biological process and provided the significance in terms of p-value in the result section. Added the details of hypo- and hyper-methylated genes and have discussed their relevance to autism in the discussion section.

**Result section:** The biological functional enrichment showed four biological functions to be significantly overrepresented. The four functions are: (i) Quantity of synapse (p-6.37E-19), (ii) Microtubule dynamics (p-6.06E-8), (iii) Neuritogenesis (p-1.68E-7) and (iv) Abnormal morphology of neurons (p-5.99E-7) (Fig 1). Among the enriched genes in the above said biological functions, about 93% were hypomethylated and 7% were hypermethylated genes. These molecular pathways are relevant to neuronal dynamics, cognition, and autism. The relevance of these biological functions is further discussed.

**5. Review Comments to the Author**

Please use the space provided to explain your answers to the questions above. You may also include additional comments for the author, including concerns about dual publication, research ethics, or publication ethics. (Please upload your review as an attachment if it exceeds 20,000 characters)

**Reviewer #1:** Current Manuscript describes the use of placental DNA methylation alterations and the early prediction of autism in full-term newborns. Autism spectrum disorder (ASD) has been previously linked with abnormal brain development and epigenetic dysfunctions during the early development stages. The authors hypothesized if placental DNA methylation changes could be used as prediction tools to elucidate the early pathogenesis of ASD. They performed genome-wide methylation analysis of 14 patients and 10 controls using placental tissue followed Ingenuity Pathway Analysis, six Artificial Intelligence (AI) algorithms including Deep Learning (DL) to investigate the predictive accuracy of CpG markers for autism detection. They identified important biological pathways and genes involved in the early fetal neurodevelopmental process that influence later cognition and social behavior. Overall, this is an excellent study that provides important insights about the biological pathways involved in ASD. The authors should address the following minor concerns to improve the quality of their manuscript.

1. The authors performed genome-wide methylation analysis using 14 patients and 10 controls. The gender of these individuals is not clear. The authors should specify this in the table and methods section.

**Author's Response:** Thank you for your advice, now we have included the details in the methods section.

2. There are few incomplete statements in the manuscript. Line 239, stated, “Further functional studies are required to explore its role in ASD”. What do you mean by further functional analysis here? A little more clarity would be helpful.

**Author's response:** The statement has been modified “However, molecular studies are required to explore its role in ASD.”

3. The authors should consider including few statements in the discussion section if the biological pathways identified in this study are druggable.

**Author's response:** We have included the statement in the discussion section (line 324-326)

4. There are multiple typos, grammatical and syntax errors that the authors should correct.

**Author's response:** We have made language corrections in the manuscript. (track changes).
